# Establishing a Centralized Virtual Visit Support Team: Early Insights

**DOI:** 10.3390/healthcare11162230

**Published:** 2023-08-08

**Authors:** James McElligott, Ryan Kruis, Elana Wells, Peter Gardella, Bryna Rickett, Joy Ross, Emily Warr, Jillian Harvey

**Affiliations:** 1 Department of Pediatrics, Medical University of South Carolina, Charleston, SC 29425, USA; 2 Center for Telehealth, Medical University of South Carolina, Charleston, SC 29425, USA; navon@musc.edu (E.W.);; 3 Department of Healthcare Leadership & Management, Medical University of South Carolina, Charleston, SC 29425, USA; harveyji@musc.edu

**Keywords:** support team, clinical support, outpatient telehealth, outpatient virtual care, telemedicine

## Abstract

Background: With the removal of many barriers to direct-to-consumer telehealth during the COVID-19 pandemic, which resulted in a historic surge in the adoption of telehealth into ongoing practice, health systems must now identify the most efficient and effective way to sustain these visits. The Medical University of South Carolina Center for Telehealth developed a Telehealth Centralized Support team as part of a strategy to mature the support infrastructure for the continued large-scale use of outpatient virtual care. The team was deployed as the Center for Telehealth rolled out a new ambulatory telehealth software platform to monitor clinical activity, support patient registration and virtual rooming, and ensure successful visit completion. Methods: A multi-method, program-evaluation approach was used to describe the development and composition of the Telehealth Centralized Support Team in its first 18 months utilizing the Reach, Effectiveness, Adoption, Implementation, Maintenance framework. Results: In the first 18 months of the Telehealth Centralized Support team, over 75,000 visits were scheduled, with over 1500 providers serving over 46,000 unique patients. The team was successfully deployed over a large part of the clinical enterprise and has been well received across the health system. It has proven to be a scalable model to support enterprise-level virtual health care delivery. Conclusions: While further research is needed to evaluate the long-term program outcomes, the results of its early implementation suggest great promise for improved telehealth patient and provider satisfaction, the more equitable delivery of virtual services, and more cost-effective means for supporting virtual care.

## 1. Introduction

**Figure 1:**
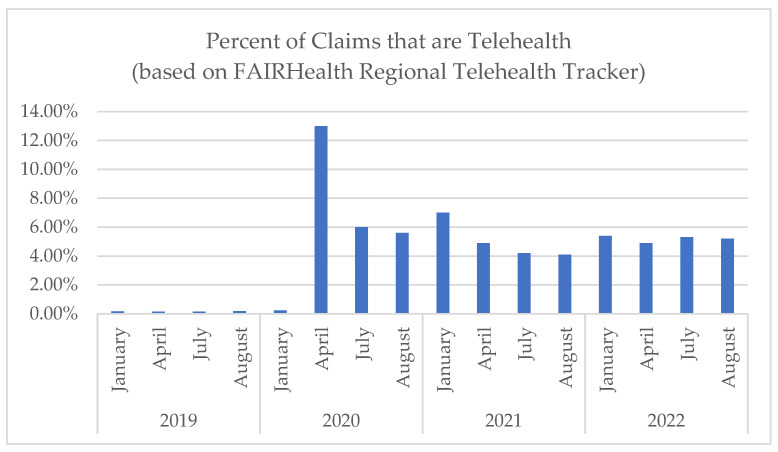
Telehealth claims as a percentage of all claims (data provided by FAIRHealth Regional Telehealth Tracker [2]).

The COVID-19 pandemic brought the removal of many barriers to direct-to-consumer telehealth, resulting in a historic surge in telehealth adoption. While volumes have declined since the early months of the pandemic, health systems continue to provide a significant percentage of care virtually. According to FAIRHealth, rates of telehealth have remained stable, around 5% of all claims throughout 2021 and 2022, which is significantly greater than pre-pandemic levels (Figure 1) [1,2,3]. These trends are likely to continue. According to the American Medical Association (AMA), “telehealth is critical to the future of healthcare [4]”. An AMA telehealth survey conducted in 2021 found that almost 70% of physicians indicated that their organization was motivated to continue to use telehealth [5]. Furthermore, more than 80% [5,6] of physicians believe that patients using telehealth have better access to care and 94% of patients who have used telehealth want to continue to have access to telehealth [6,7]. Furthermore, many of the U.S. payment and regulatory policies that allowed for the growth of telehealth during the pandemic are being made permanent at both state and federal levels [8].

With telehealth here to stay, healthcare organizations are now faced with numerous challenges as they develop care delivery models to support long term telehealth activity. First, although telehealth has the potential to expand access with fewer practice expenses [9], the work and resources associated with administering telehealth programs are still considerable and differ from those associated with in-person care (e.g., cost of administering and monitoring telehealth platforms) [9,10,11]. Identifying opportunities to streamline workflows and create efficiencies of scale will be necessary if health systems are to financially benefit from telehealth. Second, research on telehealth during the pandemic revealed disparities in access to telehealth in part due to varying levels of patient digital literacy [12,13] and disparate access to broadband and technological devices [14,15]. For all patients to have access to the benefits telehealth affords, additional focus on patient support and navigation may be needed. Finally, as health systems work to create consumer-centric models of telehealth delivery, this must be balanced with efforts to maintain provider satisfaction and ease of use, especially given the increasing rates of provider burnout [16,17]. Thus, it is of utmost importance that health systems invest in models of telehealth delivery that are efficient, effective, and equitable.

Several institutions have explored creative solutions to address these challenges. These have included volunteer medical students helping patients with low technology literacy prepare for telehealth visits [18]; recruiting medical students and other health system volunteers to assist patients over 65 in setting up a video platform and making the video connection in advance of their scheduled visit [19,20]; and offering phone-based telehealth training sessions before a telehealth visit [19,21]. Although effective, these initiatives are often time- and resource-intensive and may depend on students or volunteers [20], making them more difficult to implement and sustain at a large scale.

Centralized pools of virtual staff supporting enterprise-wide outpatient telehealth may prove to be a solution to this challenge. In late 2021, the Center for Telehealth (Center) at the Medical University of South Carolina (MUSC) developed a Telehealth Centralized Support (TCS) team. The team was deployed as MUSC rolled out a new ambulatory telehealth software platform to monitor clinical activity, support patient registration and virtual rooming, and ensure successful visit completion. Using a multi-method program-evaluation approach, this report shares early findings from the 18 months of operations including the roles and responsibilities of TCS team members, staffing ratios, communication procedures, and evolving workflows. The goal is to disseminate a promising model for health systems to support large volumes of telehealth more efficiently and equitably.

## 2. Materials and Methods

The Reach, Effectiveness, Adoption, Implementation, Maintenance (RE-AIM) framework was used to evaluate and describe the development and composition of TCS in its first 18 months [22]. RE-AIM was developed to examine the implementation, adoption and impact of translating science into practice [23]. This approach was also conducive to the rapid science approaches called for when trying to speed the dissemination of timely innovations [23,24].

We used a multi-method approach, with data sources including both qualitative and quantitative data. The project was submitted to the Quality Improvement Program Evaluation Self-Certification Tool sponsored by MUSC’s Institutional Review Board (IRB) [25]; the project was deemed to be program evaluation and thus did not require formal review. An initial focus group was conducted virtually in November 2022 by members of our evaluation team (J.H., E.W.) with leaders of the TCS implementation team, some of whom are also included as authors of this manuscript. Focus group participants included the following: the Executive Medical Director (J.M.), Director of Operations (P.G.), initial TCS managers (B.R., J.R.), and one TCS team member. The focus group was held on Microsoft Teams, lasted 49 min and followed a semi-structured interview guide examining TCS workflow, staffing model, and insight gained from those who developed as well as currently implement TCS. The focus group was recorded and transcribed within the Microsoft Teams platform. The evaluation team (J.H., E.W., R.K.) merged qualitative data from focus groups with workflows, tracking data, and program documents to develop thick descriptions of TCS staff roles, staffing ratios, inter- and intra-team communication, utilization, and lessons learned. The descriptions were developed through an iterative process and underwent several rounds of validation and feedback from the implementation team to confirm descriptions. This was achieved through an in-person, follow-up interview with two members of the implementation team (B.R., J.R.) as well as an asynchronous review of descriptions provided by other TCS implementation team members (J.M., P.G., E.W.). This validation occurred in the spring of 2023. During the interviews, respondents provided further detail on the roles of different TCS members and clarified the organization chart. Next, we incorporated program tracking and patient data from the telehealth software, electronic health record, IT support tickets, and health system dashboards. Table 1 highlights the program evaluation measures utilizing these data sources. In this paper, we highlight the descriptions developed through this multi-method process, and incorporate both qualitative and quantitative data throughout.

## 3. Results

The TCS is housed within the Health System’s Center for Telehealth. The Center for Telehealth has over 15 years of experience providing telehealth, offering over 100 unique telehealth services to over 280 sites across South Carolina. Care settings include over 45 hospitals, over 90 schools, and over 100 community clinics and other facilities. The TCS team was developed in response to pressing patient and provider needs that resulted from the rapid expansion of direct-to-patient telehealth video visits across ambulatory specialties during the pandemic. Like in-person patients, telehealth patients still require standard registration and medication reconciliation typically handled by frontline staff. Additionally, they face the added challenge of needing to navigate the telehealth platform on their personal device to begin visits. Prior to TCS, each department handled virtual patient registration differently, sometimes utilizing existing registration staff but often relying on the provider themselves to educate and problem solve with a patient, resulting in an inconsistent and inefficient process for patients and providers alike.

The virtual TCS team includes an interprofessional team of Certified Medical Assistants, Licensed Practical Nurses, and is overseen by a TCS manager at the Center. The primary goal of the TCS team is to ensure the success of patients and clinicians using telehealth for outpatient visits and to serve as a virtual clinical support to the provider. The current team’s structure that is outlined below is an optimization and maturation of the original, smaller-scale team, which included two individuals who started each day with a morning huddle and often turned to an “all-hands-on-deck” approach as described by a TCS team member to meet the needs of the patients and providers.

### 3.1. TCS Roles and Responsibilities

The roles of the TCS staff were established to support patient clinical intake following standard enterprise protocols, patient education, and technical support. For clinics engaged with the TCS model, the TCS team provides at least one audio call to each telehealth patient up to one business day prior to the patient’s telehealth visit. The purpose of the audio call is to complete the pre-visit required intake and to communicate what the patient can expect to experience from the video platform. The pre-visit required intake, which varies by specialty, can include medication reconciliation, questionnaires, and patient-reported vital signs (including blood pressure reading, weight, and blood sugar testing). The TCS team members make three attempts to reach each patient. If the TCS team member cannot reach the patient, they note this in the patient’s chart in the electronic medical record (EMR) so that the provider can decide if they would like to see the patient or if the visit must be rescheduled. Additionally, the TCS team provides support during each visit by troubleshooting technology as needed based on patient and provider needs.

A full description of each of the roles and team responsibilities are outlined in Table 2. In addition to the TCS team itself, the center and hospital system staff also provides ad hoc wraparound support to the TCS team.

**Figure 2:**
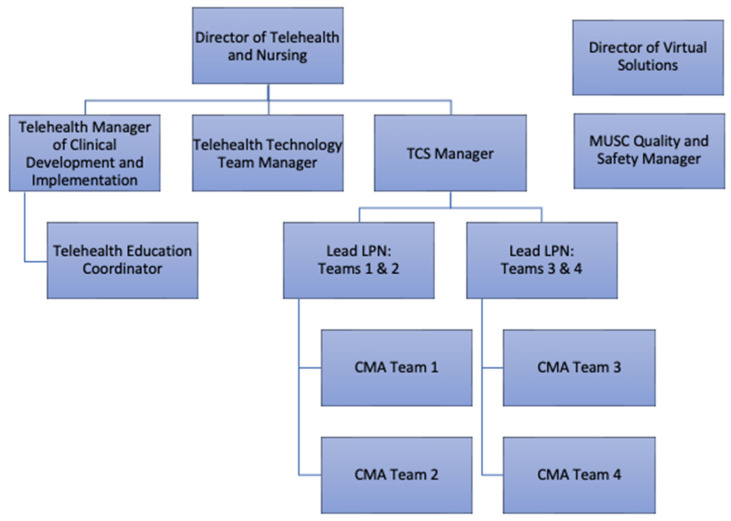
Organizational Reporting Structure of TCS Team Members. Note: Descriptions of team roles are included in Table 2.

The front-line work of TCS is carried out by CMAs, organized into four sub-teams defined by clinical specialties with oversight from two charge LPNs. The team is led by a TCS Clinical Manager who reports to the Director of Operations and Nursing in the Center. Additional ad hoc, specialized wraparound support is offered by the Center’s Manager of Clinical Development and Implementation, Telehealth Education Coordinator, Virtual Solutions Director, and technical support team as well as the health system’s Quality and Safety Manager. The organizational structure is depicted in Figure 2.

### 3.2. TCS Scope and Staffing Ratios

In the current state, TCS supports nearly all adult and pediatric primary care and specialty clinics across the MUSC Charleston campus and surrounding clinics. TCS also provides a lighter form of support focused mainly on resolving connection problems to providers affiliated with MUSC Health’s Regional Health Network, a recent expansion of the MUSC Health system to new regions in South Carolina. One current exclusion is for psychiatry which does not participate due to its large volume of virtual visits relative to in person visits and robust departmental virtual visit procedures. Additional exceptions include multi-specialty clinics with complex virtual visit workflows. Staffing ratios have fluctuated since the launch of TCS based on the onboarding of new specialties, hiring permanent TCS care team members, and obtaining additional CMA and LPN support through contracting with a staffing agency. The center used a staffing agency to help scale the team quickly while the team waited on the hospital system’s approval of the permanent TCS positions. As the center received approvals to hire TCS team members, the positions were posted, and the center has in turn hired approximately 90% of the TCS team members that were brought on through the staffing agency. The team has grown to 14 team members over the past 18 months, each supporting at least 30–40 visits per day and collectively 400–500 visits daily. Each CMA is responsible for different clinics as explained by one TCS team member, “As we continue to grow and with this last expansion, we did a staffing optimization to where we said we need to separate out into some group that way clinics know who they’re dedicated people are to go to for communication purpose, and we’re making sure that we’re having a smaller set of patients… you know, for each group to concentrate on”.

### 3.3. Communication

An essential element to the work of TCS are the Microsoft Teams asynchronous chat threads to support communication with the providers and clinical staff, and through the patients via the pre-visit phone calls.

#### 3.3.1. Provider and Clinic Staff Communication

Communication between TCS and the clinic staff are organized across the enterprise in over 150 separate asynchronous chat threads via Microsoft Teams. Additionally, a common phone number for clinic staff to call the TCS was established, though the clinic staff use the Microsoft Teams chat function most often. Each clinic has a unique chat thread, typically including participating providers, the lead nurse, and the lead patient registrar as described by a TCS team member “… there are a lot of areas [specialties] that the providers do not want to be a part of the chat. So, we kind of pivoted as we were going to the clinics to do training. We would capture their lead nurse or any lead registration folks or anybody that we knew would be watching the chats to help us communicate with the providers if they’re there in person especially… so it kind of pivoted more… to the clinic level”.

Provider use of the TCS communication threads is variable in the current state. In hybrid in-person and telehealth clinics, the providers commonly rely on their local support staff to communicate with the TCS team as described by a TCS team member, “[In] a hybrid setting, [they] have a nurse usually at their elbow that they can grab onto and utilize”. Providers who are not working in the clinic and seeing only telehealth patients on a given day use the chat function regularly as their remote clinical and administrative support as described by a TCS team member “…the providers who have a block [virtual] schedule that work from home use TCS as their clinical staff. They are more…vocal in the chats because they don’t have anyone else to help them”. The most common reason for using the Microsoft Teams asynchronous chat thread is an outreach from the provider when the patient is not in the visit as expected.

One current limitation is the inability of the TCS team to route the patient for rescheduling without further provider or clinic office staff engagement. A second challenge of the chat function is that the amount of chat traffic occurring in a single thread can be distracting, particularly for providers with infrequent virtual visits. Establishing provider-specific chat capabilities for the TCS team has proven challenging due to the volume of participating providers.

#### 3.3.2. Patient Communication

As noted previously, a major component of patient communication is the pre-visit telephone call TCS conducts with patients to conduct intake paperwork and orient the patient to the video visit. This is typically conducted the day prior to the visit or the morning of for a few clinics.

During the visit itself, TCS is regularly monitoring the telehealth technology platform dashboard of patient statuses in the TCS triage workflow to determine whether any additional patient outreach is needed. Sometimes, TCS will contact a patient via audio call if a provider is running late. Most commonly, patient outreach during a visit occurs due to a patient not having arrived to the virtual visit during the scheduled appointment. When this occurs, a TCS team member will contact the patient via an audio call and assist them in connecting to the visit. If the patient is not able to be reached or is unable to connect to the visit, the TCS team member will share with the provider or clinic staff that the visit needs to be rescheduled due to the patient not being available or not being able to make the connection in time. Patient experience data suggested this type support was critical. For example, one patient noted in their post-visit survey, “[the provider] was able to see us but we couldn’t hear so we did it over the phone after several attempts to get it correct”.

### 3.4. Utilization

**Figure 3:**
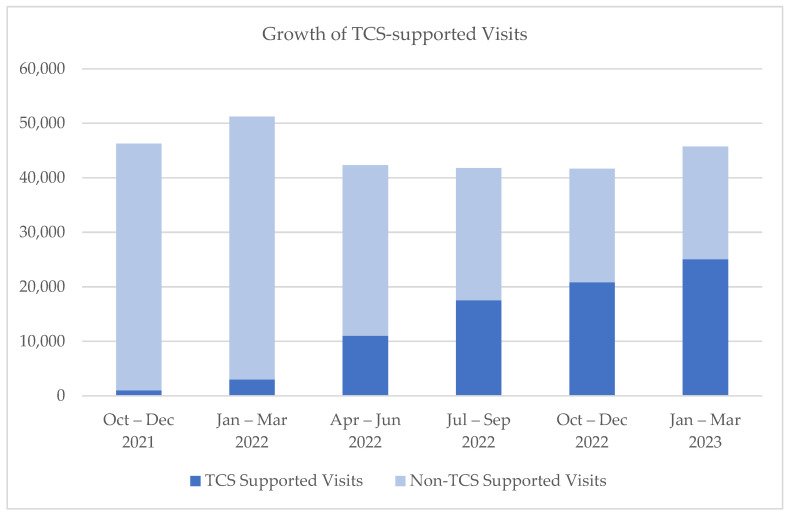
Growth of TCS-supported Visits.

**Figure 4:**
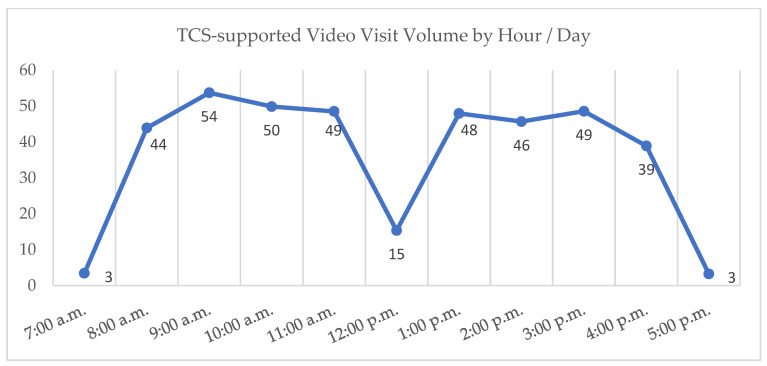
TCS volumes by hour/day.

In the 18 months since the launch of TCS in October of 2021, over 75,000 visits were completed with support from the team and using the new telehealth platform, with over 1500 providers serving over 46,000 unique patients. Figure 3 demonstrates the growth of the TCS team as represented by the number of visits supported out of all ambulatory virtual visits conducted in MUSC’s Charleston division, and Figure 4 provides a snapshot of TCS volumes of visits by hour for a typical day.

**Figure 5:**
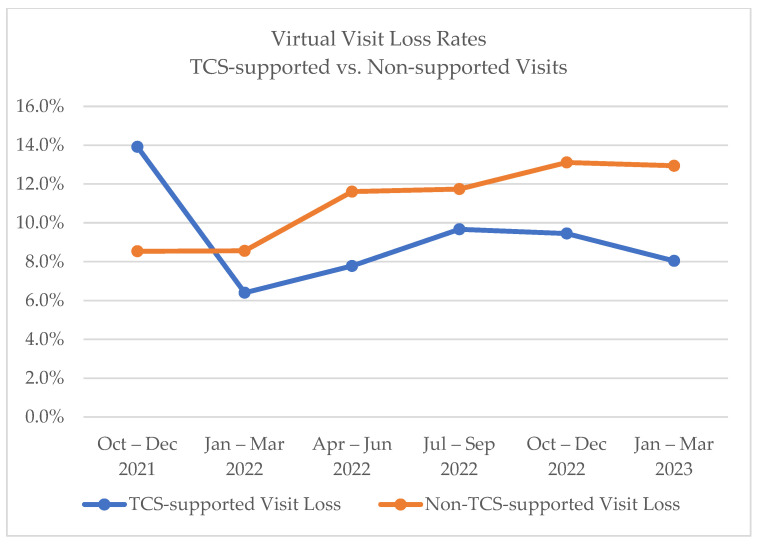
Virtual visit loss rates.

Of note, providers have had some difficulties moving to the new telehealth platform with patient triage features, as providers have become used to sending links from unintegrated solutions and relaxed oversight on compliance requirements set forth at the beginning of the COVID-19 pandemic. However, virtual visits supported by TCS have lower rates of visit loss (i.e., patient no-show or cancellation within 24 h), as compared to virtual visits not receiving support from the TCS team, as seen in Figure 5.

## 4. Discussion

To maintain high quality care in a post-pandemic world of elevated virtual care use, forward-leaning health systems must provide cost-efficient support infrastructure for virtual visits. While digital automations and patient self-directed portal use have the potential to improve virtual visit experience, innovations in the use of human workforce remain essential components of virtual care growth strategies. The TCS staffing approach has been successfully deployed over a large part of the MUSC clinical enterprise within the 18 months of operations. TCS has been well received across the health system and has become increasingly relied upon during this transition period of technology and workflow conversions related to the expiration of the Public Health Emergency.

The TCS model exemplifies the broader move in healthcare toward “systemness”, i.e., efforts made toward streamlining workflows, creating a more consistent patient experience, and leveraging centralized resources across multiple and varied departments within a health system [27,28]. Such efforts improve organizational consistency and quality and have potential to address widespread staffing shortages [29]. TCS capitalizes on these key concepts of systemness, leveraging efficiencies of scale to provide a unified, high-quality virtual care experience.

Importantly, investment in virtual visit support models, like TCS, can help mitigate the telehealth equity concerns raised during the pandemic. Dedicated telehealth support staff and flexible telehealth workflows that can adapt to patient needs have both been identified as components that can support health equity in telehealth [30]. Others have explored the potential for digital health navigators to support patients who are less digitally literate with use of patient portals, smartphone apps, wearables, and telehealth to support clinical care [31,32]. Providing direct human support through patient-centered models like TCS help prevent furthering the digital divide among underserved populations and those with digital literacy challenges.

Although successful, opportunities for improvement are considerable in the areas of communication, staffing, workflow, and technology. Currently, providers and clinic staff receive messages that they do not need through the asynchronous chat. The team is exploring opportunities for more focused communication so that providers only receive messages that are directed to them. Additionally, contract workers were initially used to build out the TCS team; however, hiring team members onto the TCS team permanently has supported retention and decreased re-training effort needs. In the area of workflow specifically, the process for re-scheduling patient appointments for those unable to connect has been identified as a challenge, as the TCS team does not have scheduling rights in the EMR. This is intentional as the center aims to ensure the TCS roles and responsibilities remain focused. Plans are underway to identify a more streamlined approach to inform clinics when a patient needs to be rescheduled.

In the area of technology, patient portal use, telehealth platform automations, and targeted digital literacy risk scoring are ongoing initiatives that have not yet been launched during the 18 months of operations. Having established a core workforce and set of procedures to support high-volume video visit use, we hope to deploy predictive analytics on digital literacy to further gain workforce efficiencies and develop additional interventions to further reduce digital care disparities. Another opportunity is increased coordination with other automations across the MUSC system to avoid messaging fatigue to the patient, as one TCS member put it: “TCS as a whole is greatly appreciated by the patients. The barrage of communication from MUSC is not”. Finally, additional layered technological interventions on our roadmap include leveraging medical record data and artificial intelligence to customize visit experiences as well as combining technology and human interventions in the post-visit space to help patients navigate their post-care follow-up and prescribed care pathways.

In summary, challenges associated with communication between TCS, the providers, and patients are present due to the complexity of a core team messaging with many disparate clinical service lines. Workflow adjustments and automations remain a consistent focus for quality improvement. Additionally, while technical difficulties are mitigated with the dedicated support, they remain a significant challenge and ongoing demand that limits the expansion of duties for the support team. Conversely, planned platform enhancements, enhanced digital literacy risk assessments and other innovations may benefit in their deployment due to the experience of the centralized team. An additional area of opportunity for improvement is the post-visit portion of the workflow, for which care coordination and patient messaging optimizations are needed.

This project has several limitations. First, we are only able to assess the first year and a half of data and are unable to assess the long-term program outcomes currently. However, as many health systems are struggling with these issues, there are valuable lessons learned from both this model and its implementation. Second, this is an examination of one health system’s program. The results and processes may not be fully generalizable to other settings and regions, but other systems may adapt our workflows to meet their individual needs.

Future areas of research will focus on the cost savings of TCS staffing as compared to both previous departmental support for telehealth and more traditional in-person support staffing. Additionally, we intend to examine the accuracy of digital risk scoring in predicting patient support needs and the impact of the TCS model on patient and provider satisfaction.

## 5. Conclusions

Finally, despite opportunities for continued improvement on workflow automations, communication methods, technology enhancements, and provider education, TCS is largely seen as a success among the MUSC enterprise due to its ability to support enterprise-level virtual health more efficiently. While further research is needed to fully evaluate the TCS model, the results of its early implementation suggest great promise for improved telehealth patient and provider satisfaction, more equitable delivery of virtual services, and more cost-effective means for supporting virtual care.

## Figures and Tables

**Table 1:** RE-AIM Framework for implementation and evaluation of the TCS Program.

RE-AIM Domain	Measures
Reach: Representativeness of participants	Visits scheduled; unique patients; patient demographics
Effectiveness: Program Outcomes	Number of Visits, Visit completion rates, patient telehealth satisfaction; equity of telehealth utilization; number/type of technical issues
Adoption: Willingness of sites/staff to use the program	Number of participating providers, staff/provider satisfaction; number/type of participating clinics; staff hire/turnover
Implementation: Degree to which the program is delivered as intended and participants’ use of the intervention	Team roles, and changes overtime; staffing ratios; communication protocols, workflow adherence and fidelity; volumes by hour; chat message volume
Maintenance: Long-term sustainability and outcomes	Cost-effectiveness, program expansion and attrition

**Table 2:** Roles and Responsibilities.

TCS Team	
Certified Medical Assistants (CMAs)	Support to Patients: Pre-visit audio call Support patients who have difficulty connecting to the visit through education, digital literacy, and technical support Support remote patient clinical intake processes Support to Providers: Monitor each clinic’s asynchronous chat, which typically includes the clinic’s lead nurse or lead patient care access coordinator Troubleshoot technology and escalate to the technical support team, if needed Serve as point-of-contact if the patient has not joined the visit Communicate with providers and clinic staff if the patient must be re-scheduled
Licensed Practical Nurses (LPNs)	Manage CMAs and design schedule to meet the needs of each specialty, which can span 12 h (7 a.m.–7 p.m.) Assign tasks to CMAs Cover gaps in CMA schedule including lunch breaks
TCS Manager	Manage and provide overall direction to team Monitor process measures Implement process improvement efforts
Ad Hoc Wraparound Support	
Telehealth Director of Operations and Nursing	Directs the clinical operations, strategic expansion of clinics using the TCS model, and supervises the TCS manager
Telehealth Manager of Clinical Development and Implementation	Collaborates with the TCS Manager in the development of new services and clinics that will be using TCS Refine education and training based on feedback from clinics
Telehealth Education Coordinator	Directly educates providers on video client and communicates back to the TCS team to share the level of support the provider may need (e.g., adding a provider to a TCS Microsoft Teams chat [26]; connecting a clinic manager to TCS) Trains new TCS team members
Virtual Solutions Director	Collaborates with vendor and requests optimizations to the platform
Technical Support Team	Provides technical support to providers and patients that has been escalated by the TCS team
Quality and Safety Manager	Meets with center leadership regularly to share data to support quality improvement efforts

## Data Availability

Limited data available on request from the corresponding author. Data are limited to focus group transcripts and administrative operations data summarized in the article itself. These data are not publicly available due to privacy.
